# Clear aligners with differentiated thickness and without attachments – A case report

**DOI:** 10.4317/jced.59618

**Published:** 2022-06-01

**Authors:** Alessandra Putrino, Maria-Rosaria Abed, Carlo Lilli

**Affiliations:** 1 DDS, Specialist in Orthodontics, PhD Researcher, Post-doc Programme in New Technologies and Law at MICHR – “Mediterranea International Centre for Human Rights Research”, Reggio Calabria; Università degli Studi di Roma “La Sapienza”; 2 DDS, Specialist in Orthodontics, PhD Researcher , Private practice in Madrid, Spain; 3 DDS, Specialist in Orthodontics , Private practice in Lodz, Poland

## Abstract

**Background:**

Most clear aligner systems use straight or scalloped gingival margin aligners that are replaced weekly and that mainly use attachments to guide many movements. Yet in the literature some studies show the effectiveness of the aligner margin extended beyond the gingival margin, and divots instead of attachments and the biological advantage given by the use of aligners with differentiated thickness.

**Material and Methods:**

A female patient (23 years old) with a pronounced proclination of the upper and lower incisors and moderate crowding who was treated with aligners weekly replaced with differentiated thickness, divots, no attachments and a straight margin beyond the gingival margin.

**Results:**

The therapy was carried out in 5 months and did not need any refinements. A total of 40 aligners were used (20 with soft thickness and 20 with hard thickness for each arch).

**Conclusions:**

Invisible aligners without attachments and with other therapeutic strategies such as divots and differentiated thickness are a valid alternative to traditional aligners that cannot be ignored.

** Key words:**Orthodontics, clear aligners, appliances design.

## Introduction

The clear aligners have characteristics that unite them such as the material they are made of (mainly polyethylene terephthalate glycol and polyurethanes), the design of the gingival margin (straight or scalloped), the presence of composite resin buttons called “attachments” that guide most of the movements, the “stripping” procedure that is the reduction of interproximal enamel (IPR). Many brands of aligners also share the same protocol which involves an average replacement of aligners every two weeks (1). The evolution of aligners in recent times has led many brands to introduce the integration of auxiliary systems such as elastic and mini-screws to expand therapeutic possibilities (2-4). On the other hand, however, also thanks to the perception of the aligners by patients as a choice that has not brought any benefit in terms of aesthetics and comfort (5,6), some brands are adopting and developing solutions that are aimed at simplifying the systematics with aligners with an increase in aesthetics and comfort without sacrificing effectiveness (7). The differentiated thickness also takes up a well-known concept of orthodontic biomechanics on the basis of which the modulation of light and moderate forces produces a better therapeutic effect than a constant force (8,9). Based on these considerations, we present the clinical case of a patient treated with a clear aligners system (Sorridi®, Tecnologia Dentale Spa, Latina, Italy) without attachments, which uses a straight gingival margin design and alternates two thicknesses of aligners through the weekly replacement. The results obtained with this system are encouraging. The chair sessions were short because there was no need for bonding of attachments (and their eventual rebonding in case of detachment). Furthermore, the possibility of using aligners with differentiated thicknesses to be alternated weekly guarantees greater stability and continuity in the treatment by reducing the overall wear of each aligner.

## Case Report

**Figure 1:**
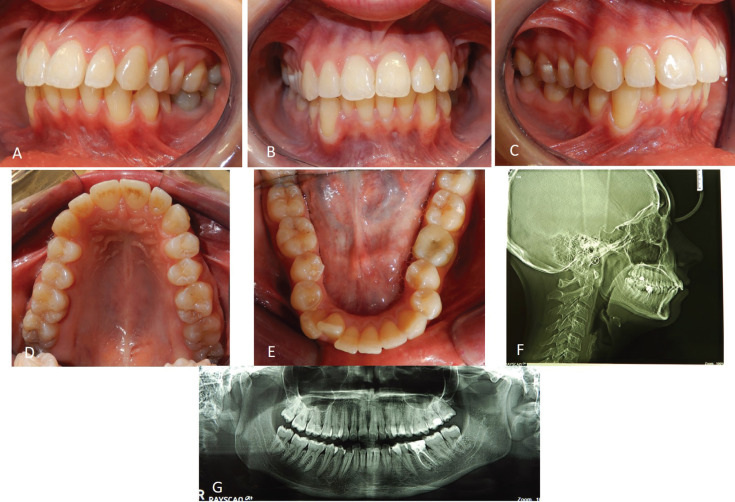
Intraoral photos of the patient before the treatment (A-E) and initial radiographs (F, G).

**Figure 2:**
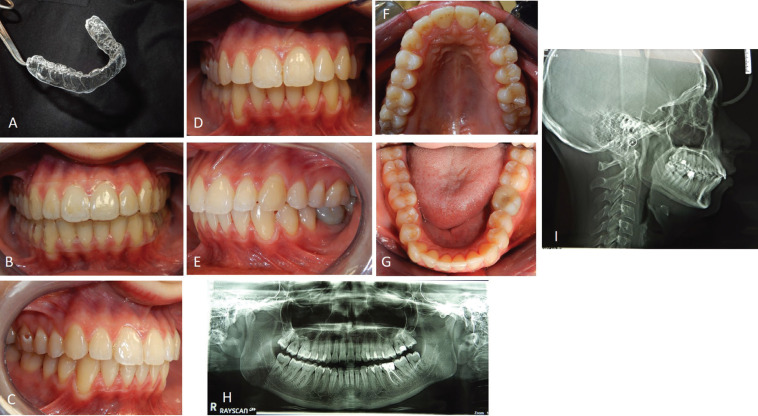
Appearance of a SORRIDI® clear aligner (A). The patient wearing both “SORRIDI” clear aligners (B). Post-treatment intraoral photos of the patient (C-G). Post-treatment radiographs of the patient (H, I).

**Figure 3:**
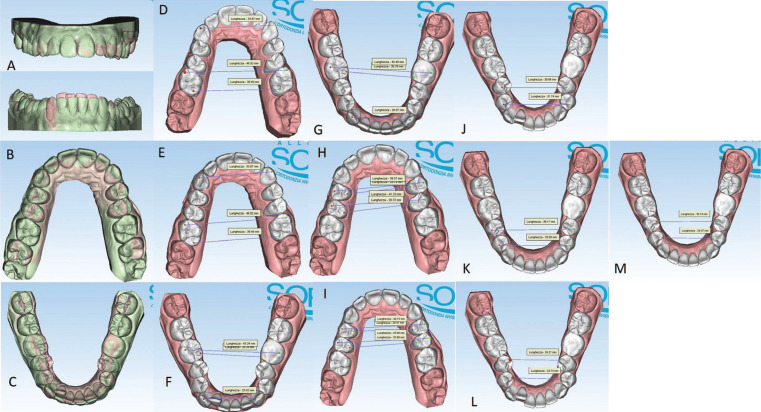
Significant movements from orange to green colour (A-C) and pre and post treatment distances (translated from italian language “lunghezza”) between the cusps of the canines, mesial buccal cusps of first upper molars, disto-oral cusps of first upper molars, centro-vestibular cusps of first lower molars, mesio-oral cusps of lower first molars, buccal and oral cusps of the first and second premolars (D-M).

In September 2021, a 23-year-old female patient requested an orthodontic evaluation for possible therapy with clear aligners. The patient was not available to receive aligner treatments that included attachments. She considered them unaesthetic and uncomforTable on the basis of friends’ experiences. The patient residing many kilometers away from the dental clinic and very busy for work despite the pandemic did not want to give up an orthodontic treatment which, however, was limited as much as possible in discomfort, check-ups and unsightliness. Before satisfying her needs and expectations, the clinical situation was assessed and orthopantomography and teleradiography in lateral-lateral projection were requested. The patient, who had never previously undergone orthodontic therapy, was in permanent dentition, first molar class both right and left, first canine class on the right and second on the left side (Fig. 1A-E). There was contraction of the upper arch in both posterior lateral sectors (from first premolar to second premolar) and moderate crowding and contraction of the lower arch with lingual orientation and rotation of the second premolars on both sides. The right lower lateral incisor was lingually displaced. The left lower first molar presented a large restoration with underlying endodontic therapy visible on the orthopanoramic radiograph (Fig. 1G). Also, the second upper left molar showed the presence of a composite resin restoration. The right upper first molar showed mild enamel discoloration of no pathological significance. There was no caries or other injuries, except the need for professional scaling (Fig. 1F). From the cephalometric analysis performed using the ViewBox© Software Version 3.1.1.14 (dHAL Software, 6 Menandrou Street, Kifissia 14561, Greece) on the initial lateral teleradiography (Fig. 1, Table 1), the patient was mainly a hyperdivergent (SN to GoGn35,5°; SN to GoMe 34,4°) Class II malocclusion (ANB 4,6°), with upper and lower incisors pro-inclined (Interincisal Angle 107,8°; Upper Incisor to NA 28,5°; Lower Incisor to NB 39,2°). The double impression technique in silicone material (Elite HD+ Putty Soft Normal and Elite HD+ Super Light Body, Zhermack SpA, Badia, Italy) allowed to obtain virtual models and setup based on these therapeutic goals: lower crowding resolution, expansion of the posterior lateral sectors in both the dental arches, reduction of rotation of the rotated dental elements, reduction of the pro-inclination. The setup obtained indicated the need to subject the patient to the treatment with a succession of 20 upper and 20 lower aligners for a total of 10 movements per arch (for a total period of 5 months of treatment). The Sorridi® system uses two aligners in PET-G with different thicknesses defined as “soft” and “hard” for each programmed movement. The soft aligner has a thickness of 0.06 mm and is used first for a week. Then the hard thickness of 0.08 mm is used for another week until the next soft type aligner. In this way the two thicknesses are alternated weekly. For each movement there are two pairs of soft and hard aligners, respectively. The gingival margin design is not scalloped but straight beyond the gingival zenith above 2 mm (Fig. 2A,B). Before applying the first and second soft aligner (respectively in the first and third week of treatment), the programmed interproximal reduction (stripping) or IPR was carried out. In the upper dental arch, a stripping of 0.20 mm was performed mesially and distally to the first and second premolars. In the lower dental arch, a stripping of 0.10 mm was performed mesially and distally to the central incisors, of 0.20 mm mesially and distally to the canines, the first and second premolars and the first and second molars. The procedure was performed using a reciprocating contra-angle handpiece with its interproximal reduction set (Intensiv Swingle Professional Kit, WG-69 LT Ortho PROF, W&H Synea, Intensiv©, Montagnola, Switzerland). Starting from the fifth hard aligner, a pair of divots were pre-inserted to torque mesially the upper right canine in the V1-L1 conFiguration, i.e., on the facial and lingual distal surface of the tooth on the gingival. At the end of the treatment no further refinements were judged particularly urgent (Fig. 2C-G). Orthodontic retainers were applied. Post treatment radiographs were requested and cephalometric analysis was performed again too (Fig. 2H,I, Table 1). The Interincisal Angle increased its value (110,7°) and the overjet and overbite values improved as well (3,5 and 3 mm respectively). Incisors pro-inclination decreased (Upper Incisor to NA 26,1°; Lower Incisor to NB 38,6°). On the Allineatori Sorridi® viewer, which is a free of charge module of the Maestro 3D Ortho Studio software (Maestro 3D©, AGE Solutions S.r.l., Pontedera- Pisa, Italy) released by the company to every dentist or orthodontist who carries out a treatment with their aligners, the superimposition of the virtual models between the initial and final impressions made it possible to evaluate the extent of the movements actually obtained (Fig. 3A-C). Furthermore, the following distances were measured before and after the orthodontic treatment: between the cusps mesio-buccal of the first upper molars; between the disto-palatal cusps of the same; between the mesio-lingual cusps of the lower first molars and between the disto-buccal cusps of the same (the distal cusp of the right first lower molar was scarcely detailed due to the conservative restoration); the distance between the cusp top of the upper canines (Fig. 3D-G). The intermolar and intercanine distances of the upper dental arch (respectively 46.92 mm, 39.46 mm and 33.87 mm) remained unchanged as expected from the virtual setup and reported in the detailed movements sheet (Fig. 3D,E). In the lower arch, on the other hand, the intermolar and intercanine distances have changed as expected by the virtual setup. The lower intercanine distance increase of 0.65 mm, the intermolar distances increase of 2.25 mm between the disto-buccal cusps and increased of 2.44 mm between the mesio-lingual cusps (Fig. 3F,G). The distances between the buccal cusps and between the palatal cusps of the first and second upper premolars also increased, with a millimeter difference of 0.58 and 2.76 for the distances between the buccal cusps of the first and second premolars, respectively, and a difference of 1.27 and 2.97 mm for the distances between the palatal cusps of first and second premolars (Fig. 3H,I). The important variation of these values must be attributed to the expected coronal-root torque movements and derotation. The lower premolars also underwent significant displacements with an important variation in the distances between the buccal and lingual cusps related to the correction of rotations and coronal-root torque of these elements. The distances between the buccal cusps of the lower first and second premolars increased by 7.49 and 1.31 mm, respectively, while the distances between the lingual cusps increased by 5.93 and 1.24 mm (Fig. 3J-M). The only pair of divots to guide the mesial torque pre-inserted on the hard aligners (from the fifth) in correspondgnce with the right upper canine did not affect the intercanine distance. On the lower anterior group, not only the vestibularization of the right lateral incisor is remarkable, but also the degree of intrusion between -0.26 mm and -0.11 mm (Fig. 3A).

**Table 1:**
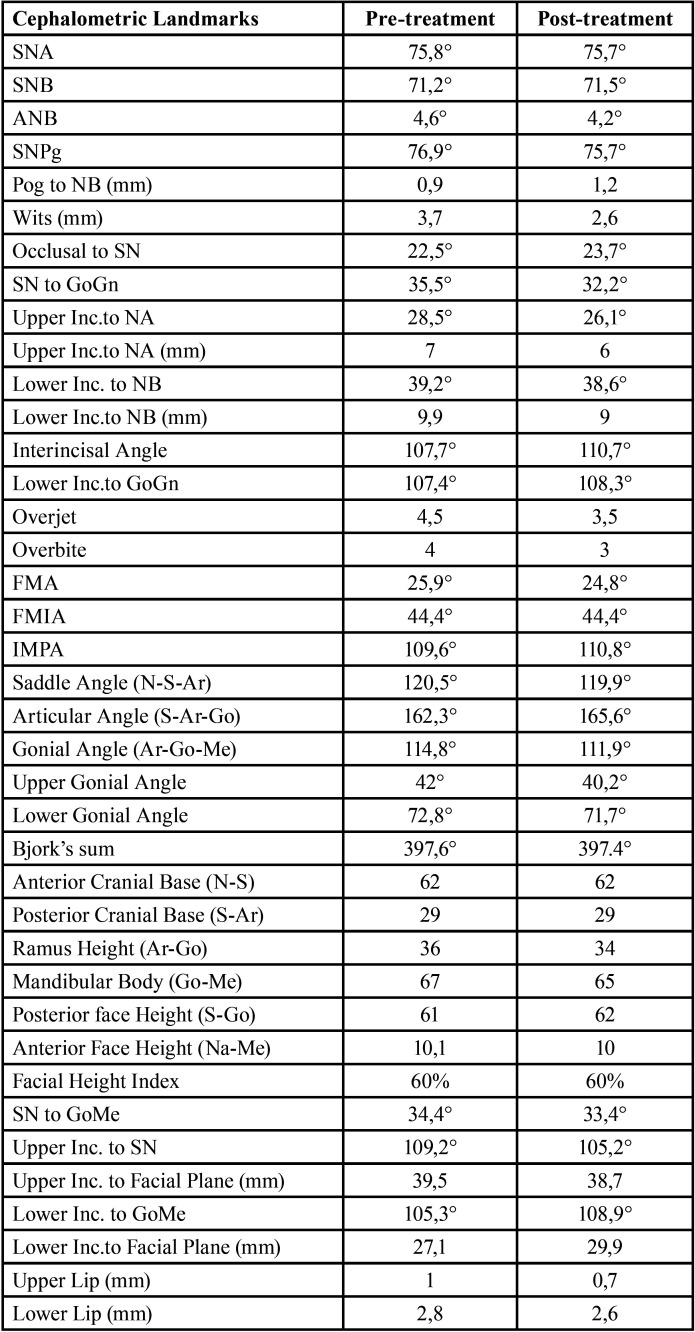
Cephalometric analysis results of the pre-treatment and post-treatment lateral teleradiographies.

## Discussion

There has been much debate in the scientific community about clear aligners ability to obtain satisfactory results even in the case of therapies that require complex tooth movements (1-5). Much of the scientific production in our possession on clear aligners derives from the use of perhaps the best known and most widespread system in the world which is Invisalign® (1,7). Experiences with different systems not only as a commercial brand but also and above all for different characteristics and protocols find it more difficult to emerge in the literature. Yet the global commercial offer of clear aligners is very broad and corresponds to the development of biomechanical strategies slightly or profoundly different from Invisalign® (7). Few studies, for example, refer to the contribution that an aligner gingival margin makes to therapy. So, we know that by definition an aligner can have a scalloped edge (like Invisalign® and in most other aligners on the market), straight above the gingival zenith, or straight but extended well 2 mm beyond the gingival zenith, but we don’t have many studies in this regard that justify the choice of one type or the other (10). The extension of the straight edge lies in the ability of the aligner with the wider margin to become more retentive (11). This is particularly important when using, as in the case presented here, aligners without attachments with a retentive function. Extending the aligner beyond the gumline with a non-scalloped design reduces yield strength and improves aligner-to-tooth adhesion (10,11). This is also an important factor. In fact, the application of attachments introduces possible elements of instability and inefficiency of the tooth-aligner system because it is subject to imperfections (operator-dependent) and detachments (12). Yet studies continue to emerge in the literature that address the issue of attachments from various points of view and there are few studies that highlight the potential of aligner systems without these resin buttons in favor of other solutions such as divots or dimples (13,14). These dimples impressed on the surface of the aligners, depending on the configurations, can be retentive or guide even moderately complex movements of tip, torque and translation (15). They are invisible on the aligner surface when worn. The presence of divots pre-inserted by the company, which, if necessary, can be further activated or deactivated through the common divot pliers, increases the precision of the movements and reduces operator errors, as happens for attachments. A recent study highlights the ability of a single pair of divots to bring a dental element back into the bone thickness without the need to resort to other strategies (15). Yet the modulation of light forces to induce orthodontic movements in biological respect of the dento-periodontal unit have been well known in the literature for a long time and continue to be considered valid (7-9). Many studies analyze the physical and mechanical characteristics of the materials most used to produce transparent aligners but few analyze the issue relating to the use of differentiated thicknesses (1,7,15). The results of this clinical case should encourage the knowledge and use of simplified systematics that in an efficient and predictable way, probably due to the type of protocol used, allow to reach the planned results without further refinements. Nonetheless, these results need to be evaluated on larger and more diversified samples from a clinical point of view to have a more complete view of the clinical indications and any limitations of applicability but should be a stimulus to continue researching in the field of orthodontics with aligners.

## References

[1] Upadhyay M, Arqub SA (2022). Biomechanics of clear aligners: hidden truths & first principles. J World Fed Orthod.

[2] Patterson BD, Foley PF, Ueno H, Mason SA, Schneider PP, Kim KB (2021). Class II malocclusion correction with Invisalign: Is it possible?. Am J Orthod Dentofacial Orthop.

[3] Liu L, Zhan Q, Zhou J, Kuang Q, Yan X, Zhang X (2021). Effectiveness of an anterior mini-screw in achieving incisor intrusion and palatal root torque for anterior retraction with clear aligners. Angle Orthod.

[4] Cetta CN, Kaye RA (2019). A Reimagined Button for Elastic Attachment to Clear Aligners. J Clin Orthod.

[5] Gao M, Yan X, Zhao R, Shan Y, Chen Y, Jian F (2021). Comparison of pain perception, anxiety, and impacts on oral health-related quality of life between patients receiving clear aligners and fixed appliances during the initial stage of orthodontic treatment. Eur J Orthod.

[6] Thai JK, Araujo E, McCray J, Schneider PP, Kim KB (2020). Esthetic perception of clear aligner therapy attachments using eye-tracking technology. Am J Orthod Dentofacial Orthop.

[7] Putrino A, Barbato E, Galluccio G (2021). Clear Aligners: Between Evolution and Efficiency-A Scoping Review. Int J Environ Res Public Health.

[8] Reitan K (1970). Evaluation of orthodontic forces as related to histologic and mechanical factors. SSO Schweiz Monatsschr Zahnheilkd.

[9] Consolaro A, Consolaro RB (2018). There is no pulp necrosis or calcific metamorphosis of pulp induced by orthodontic treatment: biological basis. Dental Press J Orthod.

[10] Cowley DP, Mah J, O'Toole B (2012). The effect of gingival-margin design on the retention of thermoformed aligners. J Clin Orthod.

[11] Robertson L, Kaur H, Fagundes NCF, Romanyk D, Major P, Flores Mir C (2020). Effectiveness of clear aligner therapy for orthodontic treatment: A systematic review. Orthod Craniofac Res.

[12] Weckmann J, Scharf S, Graf I, Schwarze J, Keilig L, Bourauel C (2020). Influence of attachment bonding protocol on precision of the attachment in aligner treatments. J Orofac Orthop.

[13] Putrino A, Caputo M, Giovannoni D, Barbato E, Galluccio G (2020). Impact of the SARS-Cov2 Pandemic on Orthodontic Therapies: An Italian Experience of Teleorthodontics. Pesqui. Bras Odontopediatria Clín Integr.

[14] Mencattelli M, Donati E, Cultrone M, Stefanini C (2015). Novel universal system for 3-dimensional orthodontic force-moment measurements and its clinical use. Am J Orthod Dentofacial Orthop.

[15] D'Alessandro AC, D'Antò V, Razionale AV, Allesandri-Bonetti G (2020). Integrating CBCT and virtual models for root movement with clear aligners. J Clin Orthod.

